# DEPRESS: Dataset on Emotions, Performance, Responses, Environment, and Satisfaction during COVID-19

**DOI:** 10.1038/s41597-026-06682-w

**Published:** 2026-02-02

**Authors:** Xingtong Guo, Angela C. Incollingo Rodriguez, Chao Wang, Elke A. Rundensteiner, Shichao Liu

**Affiliations:** 1 Civil, Environmental, and Architectural Engineering, Worcester Polytechnic Institute, 100 Institute Road, Worcester, MA 01609 USA; 2 Psychological and Cognitive Sciences, Worcester Polytechnic Institute, 100 Institute Road, Worcester, MA 01609 USA; 3 Psychotic Disorders Division, McLean Hospital, Harvard Medical School, Longwood Ave, Boston, MA 02115 USA; 4 Computer Science, Worcester Polytechnic Institute, 100 Institute Road, Worcester, MA 01609 USA; 5 Data Science, Worcester Polytechnic Institute, 100 Institute Road, Worcester, MA 01609 USA

**Keywords:** Risk factors, Education

## Abstract

COVID-19 posed a significant threat to the mental health of the population in general and college students in particular, severely disrupting their daily routines due to protective measures and lockdown policies. The abrupt transition from in-person to online learning further introduced uncertainty regarding academic performance. To comprehensively assess the impacts of the pandemic on college students, this study collected longitudinal data from June 2020 to June 2021, involving 184 undergraduate students at Worcester Polytechnic Institute. The dataset includes demographic and socioeconomic status information of participants, measures of mental health outcomes, online student engagement, computer and Internet performance, daily activity diary, general indoor environment satisfaction, Fitbit data, sensor measured indoor environment quality, facial expression, and GPA. To our best knowledge, this dataset is also the first dataset that covers multimodal assessment of mental health outcomes, online learning, and potential influencing variables during COVID-19. Data was gathered through online surveys, video recordings, IoT indoor environmental sensors, and Fitbit wristbands.

## Background & Summary

In May 2020, the World Health Organization (WHO) officially declared COVID-19 as a pandemic^1^, prompting governments worldwide to implement measures to protect their citizens. Quarantine emerged as one of the most widely adopted policies, significantly disrupting daily routines. In response, most universities suspended in-person classes and transitioned to online instruction. While online learning is not new and its effectiveness has been explored in previous studies^2^, it was typically considered a supplementary tool in education before the pandemic. Meanwhile, due to the quarantine, students spend more time in their apartment or house where the indoor environment was not specifically designed for learning. How the learning performance may be affected by the bedroom indoor environment has not been well studied.

During the pandemic, mental health emerged as one of the severe issues that threatened students’ learning and well-being. Mental health problem had been identified already as a critical problem prior to the pandemic with around one in five young adults having been diagnosed with a mental health issue^3^. This situation was exacerbated due to the pandemic and the restrictive policies it brought, which led to young adults facing increased uncertainty in academic success, future career prospects, and social life^4^.

The aim of this study was thus to comprehensively understand how college students’ mental health and learning were affected by the pandemic over time as pandemic-related measures were rolled out and increasingly impacted daily life. This paper presents a longitudinal dataset, DEPRESS (Dataset on Emotions, Performance, Responses, Environment, and Satisfaction of Students), which covers mental health states, indoor environment quality, learning performance, and daily routine. DEPRESS was collected from June 2020 to June 2021 containing a rich variety of modalities of 180 undergraduate students from Worcester Polytechnic Institute (WPI). We recorded the demographic and socioeconomic status information of the participants; measures of mental health outcomes; online student engagement (OSE); computer and Internet performance; daily activity diary; general indoor environment satisfaction; Fitbit data; sensor-measured indicators of indoor environmental quality (IEQ), including CO₂, particulate matter (PM_2.5_), lighting, noise, and Volatile Organic Compounds (VOCs); general indoor environmental satisfaction; facial expression data during online courses; and academic transcripts to calculate Grade Point Average (GPA).

This study enrolled 184 participants. Among them, 152 completed daily activity diary surveys, 163 responded to mental health assessments, OSE, and computer and internet performance surveys, and 126 provided synced Fitbit data. Indoor environmental quality (IEQ) data were collected using AWAIR sensors from the bedrooms of 42 participants. This longitudinal dataset enables us to examine how college students adapted to the sudden pandemic outbreaks and how their mental health and learning performance evolved during such events.

To the best of our knowledge, this is the first dataset to capture students’ satisfaction with the physical environment while learning outside the classroom. Its uniqueness also lies in the inclusion of IEQ measurements collected via IoT sensors. Moreover, the dataset enables the analysis of students’ emotional states during online courses through extracted facial expressions, as well as investigations into how IEQ influences emotional states and mental health.

## Methods

### Data collection

**Fig. 1:**
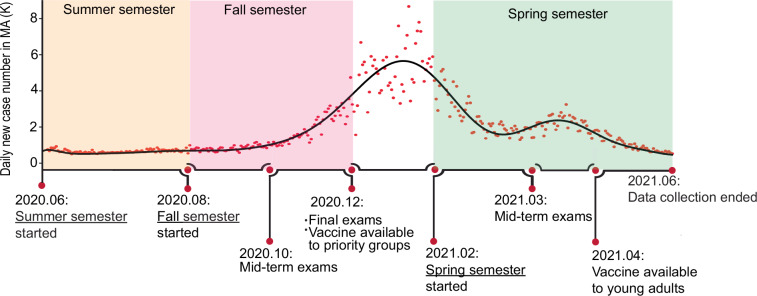
Timeline of major events during the study period and daily new COVID-19 case number in Massachusetts^22^. More than 68.6% of the participants were in Massachusetts during the study.

This study utilized longitudinal data gathered from undergraduate students at Worcester Polytechnic Institute (WPI), Massachusetts, from June 2020 to May 2021, spanning three semesters and thus composed of three different cohorts: (1) Summer Semester (June–August 2020): All students attended classes online while residing at home. (2) Fall Semester (Late August–December 2020): Students could opt for on-campus living, which included routine COVID testing. Instruction was predominantly online, with some courses offered in a hybrid format. (3) Spring Semester (February–May 2021): Most students lived on campus and participated in routine COVID testing. Most classes remained online, supplemented by some hybrid course offerings. To reduce the burden on participants and maintain a consistent data collection duration across individuals, each participant joined only one cohort. This design reduces individual workload, minimizes dropout, and helps preserve the reliability of the longitudinal patterns observed across the study. Figure 1 provides the major events that happened within the survey span based on the academic calendar of WPI.

During the study, participants were asked to fill out a series of surveys. As for the mental health outcomes survey, participants completed the Positive and Negative Affect Schedule (PANAS, scale from 20 to 100)^5^, the four-item Perceived Stress Scale (PSS, score scale from 0 to 4)^6^, the ten-item Center for Epidemiological Studies-Depression Scale (CES-D, score scale from 0 to 30)^7^ with a clinical cut-off of 10^8^, indicating the presence of depressive symptoms to a degree that may require professional help. The State-Trait Anxiety Inventory^9^ (STAI, score scale from 20 to 80), and the nineteen-item online student engagement (OSE, scale from 0 to 76)^10^.

To understand the change of students’ daily routines during the pandemic, we adapted the daily activity survey from Richard *et al*.^11^. Students reported the duration of time spent on various activities, including school-related (e.g., attending online classes, reading course materials), social (e.g., phone or in-person conversations), and entertainment (e.g., listening to music, watching TV). We also collected self-reported information on students’ demographic features, socioeconomic status, and satisfaction with internet speed and computer hardware such as central processing unit (CPU) and speaker. We required students to complete the demographic features and socioeconomic status surveys before officially participating in the study; however, we still allowed them to proceed with the study even if they had not filled it out.

In addition to the survey data, participants were asked to wear a Fitbit wristband (Inspire 2, USA) to collect sleep patterns, heart rate, calories burned, and daily activity metrics including walking distance, and step count. Some participants also agreed to install AWAIR sensors (OMNI 1, USA) in their bedrooms to monitor IEQ. Additionally, a subset of participants consented to recording only their own facial expression videos while attending online courses. The number of participants contributing to each type of measurement is summarized in Table 1.

**Table 1:** Summary of collected data sources and the number of participants contributing to each data type.

Measure type	Measurement description	2020 Summer (N, %)^1^	2020 Fall (N, %)^1^	2021 Spring (N, %)^1^	Frequency
Survey-based	Demographic and socioeconomic status	36 (75.0%)^2^	83 (85.5%)^2^	65 (64.6%)^2^	Once
	PSS, PANAS	24 (83.0%)	77 (68.0%)	62 (75.3%)	Weekly
	CES-D, STAI	24 (97.4%)	77 (88.7%)	62 (94.4%)	Monthly
	OSE, computer and internet performance	24 (83.0%)	77 (68.0%)	62 (75.3%)	Weekly
	Daily activity diary	25 (55.1%)	73 (51.8%)	54 (76.7%)	Daily
	General indoor environmental satisfaction	24 (76.7%)	77 (83.6%)	62 (68.3%)	Weekly
Sensor-based	Fitbit	14 (NA)	64 (NA)	48 (NA)	1-min
	IEQ (by AWAIR)	11 (NA)	15 (NA)	16 (NA)	15-min
Video-based	Facial expression	0 (NA)	39 (100%)	45 (100%)	At least once
Academic records	Transcript	36 (38.9%)^2^	83 (50.6%)^2^	65 (35.4%)^2^	Once

Note 1: N indicates the number of participants contributing at least one valid observation in the corresponding semester; % denotes the completion rate. Completion rate was not applicable (NA) for continuous sensor-based data, since 100% data coverage was neither expected nor feasible by design. For example, Fitbit wear time varied naturally across participants, and gaps in data primarily reflect real-world usage constraints rather than missingness relative to a fixed target. Note 2: For the one-time survey, N represents the total number of enrolled participants and % denotes the completion rate. The number of participants contributing data can be estimated by multiplying N with the completion rate.

Because different mental health outcomes fluctuate at different temporal scales, the data were collected with varying frequencies. Depression and anxiety tend to change more gradually, so these measures were administered monthly^12^. In contrast, positive and negative affect schedule, stress can vary more rapidly; therefore, the corresponding surveys was conducted weekly^13^.

### Participants and recruitment

**Fig. 2:**
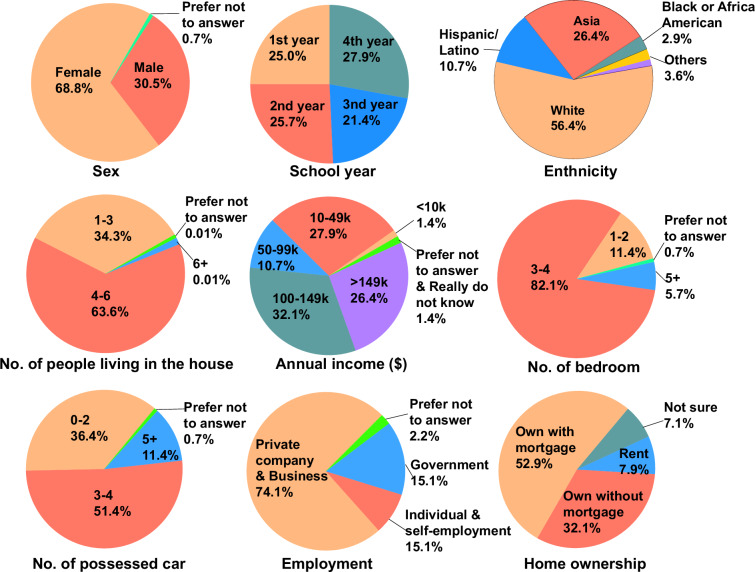
Summary of demographic features and social economic status of participants (n = 140).

Undergraduate students who had taken at least one online course during the pandemic were eligible. All participants provided signed consent before completing any surveys or measurements and were subsequently assigned a unique ID to replace their real name. Consenting participants were compensated based on task adherence. To enroll participants, we posted the study on the Student Government Association’s Facebook account, sent emails to undergraduates, and asked faculty to advertise the study in their classes. In the end, we enrolled 184 students across Summer 2020 (n = 36), Fall 2020 (n = 83), and Spring 2021 (n = 65) cohorts. At the start of the study, participants were invited to complete the demographic and socioeconomic questionnaire; completion was optional, and they can skip any questions they feel uncomfortable with. Detailed demographic features of the participants are summarized in Fig. 2 (n = 140, missing: 44). Fitbit wristbands and AWAIR sensors were picked up or shipped to the participants before their participation.

### Ethics approval

This study was conducted with the approval by the Institutional Review Board (20-0656) of Worcester Polytechnic Institute. All participants provided informed consent prior to participation. Participants received a $30 VISA gift card at the end of their cohort and were allowed to keep the Fitbit as compensation. Participation was voluntary, and refusal to participate did not result in any penalty.

The dataset released in this paper is fully anonymized, with all direct identifiers removed and only indirect, non-identifiable variables retained. In accordance with IRB guidance, the anonymized dataset does not contain information that can be used to identify individual participants, and therefore its public sharing is permitted under the approved protocol.

### Data pre-processing

For the PANAS survey, each response option was translated from 1–5, with 1 meaning “very slightly or not at all” and 5 meaning “extremely.” The subject’s positive affect score was determined by summing the response scores of the items 1, 3, 5, 9, 10, 12, 14, 16, 17 and 19, while the negative affect score corresponds to summing the response scores of the 2, 4, 6, 7, 8, 11, 13, 15, 18 and 20^5^.

For the PSS-4 survey, responses to its questions were coded based on five options. For questions one and four, this coding was as follows: “Never” = 0; “Almost Never” = 1; “Sometimes” = 2; “Fairly Often” = 3; and “Very Often” = 4. For questions two and three, this scoring was reversed, with responses coded as: “Never” = 4; “Almost Never” = 3; “Sometimes” = 2; “Fairly Often” = 1; and “Very Often” = 0. Finally, the average value of the coded responses across all four questions was calculated to determine the PSS-4 score^14^.

For the CES-D survey, responses were coded as follows: “Rarely or None of the Time” = 0; “Some or a Little of the Time” = 1; “Occasionally or a Moderate Amount of Time” = 2; and “Most or All of the Time” = 3. For items five through eight, this scoring was reversed (e.g., “Most or All of the Time” became 0 instead of 3 for these specific items, with other responses similarly adjusted). Finally, the sum of these coded values across all ten items was calculated to determine the total score^15^.

For the STAI state anxiety survey, responses were coded as follows: “Not at all” = 1, “Somewhat” = 2, “Moderately so” = 3, and “Very much so” = 4. This coding was applied to items 3, 4, 6, 7, 9, 12, 13, 14, 17, and 18. For items 1, 2, 5, 8, 10, 11, 15, 16, 19, and 20, the coding was reversed. Finally, the sum of all items was calculated^9^.

For the OSE survey, responses were coded as follows: “Not at all characteristics of me” = 0; “Not really characteristic of me” = 1; “Moderately characteristic of me” = 2; “Characteristic of me” = 3; “Very characteristic of me” = 4.

For the general environmental satisfaction survey, thermal sensations were coded from −3(cold) to 3 (hot) and satisfaction related questions were coded as: −3 (very dissatisfied) to 3 (very satisfied). Interference or enhancement of indoor environment factors with learning were coded as: “Significantly interferes” = −3; “Interferes” = −2; “Somewhat interferes” = −1; “Neither interferes nor enhances” = 0; “Somewhat enhances” = 1; “Enhances” = 2; “Significantly enhances” = 3. The amount of outdoor air and daylight (natural light) were coded as: “Far too little” = −3; “Moderately too little” = −2; “Slightly too little” = −1; “Neither too much nor too little” = 0; “Slightly too much” = 1; “Moderately too much” = 2; “Far too much” = 3. Reported learning enhancement: “Decreased 20% or more” = −3; “Decreased 10%” = −2; “Decreased 5%” = −1; “Neither increased nor decreased” = 0; “Increased 5%” = 1; “Increased 10%” = 2; “Increased 20% or more” = 3. Students’ GPAs were coded with 3 for A, 2 for B and 1 for C that was the lowest possible grade in this study. There are no pluses and minuses grades for each class.

For the computer and internet performance survey, the satisfaction-related questions were coded as: −3 (very dissatisfied) to 3(very satisfied). The download and upload speed were filled in by the participants.

For the daily activity time diary survey, responses were coded as follows: “0 min” = 0, “15 min” = 1, “30 min” = 2, “45 min” = 3, “more than 45 min” = 4. All detailed coding schemes for the survey responses are summarized in Table 2.

**Table 2:** Numerical coding for corresponding survey measures.

Survey	Items	Response (coding)
PANAS	All items	Very slightly or not at all (1)
		A little (2)
		Moderately (3)
		Quite a bit (4)
		Extremely (5)
PSS-4	Q1, Q4 (Reverse for Q2, Q3)	Never (0)
		Almost never (1)
		Sometimes (2)
		Fairly often (3)
		Very often (4)
CES-D	All items	Rarely or None of the Time (0)
		Some or a Little of the Time (1)
		Occasionally or a Moderate Amount of Time (2)
		Most or All of the Time (3)
STAI	Q3, 4, 6, 7, 9, 12, 13, 14,1 7, 18 (Reverse for Q1, 2, 5, 8, 10, 11, 15, 16, 19, 20)	Not at all (1)
		Somewhat (2)
		Moderately so (3)
		Very much so (4)
OSE	All items	Not at all characteristics of me (0)
		Not really characteristic of me (1)
		Moderately characteristic of me (2)
		Characteristic of me (3)
		Very characteristic of me (4)
Thermal sensations	All items	Cold (−3)
		Cool (−2)
		Slightly cool (−1)
		Neutral (0)
		Slightly warm (1)
		Warm (2)
		Hot (3)
Satisfaction related survey	All items	very dissatisfied (−3)
		Dissatisfied (−2)
		Slightly dissatisfied (−1)
		Neutral (0)
		Slightly satisfied (1)
		Satisfied (2)
		very satisfied (3)
Interference or enhancement of indoor environment factors	All items	Significantly interferes (−3)
		Interferes (−2)
		Somewhat interferes (−1)
		Neither interferes nor enhances (0)
		Somewhat enhances (1)
		Enhances (2)
		Significantly enhances (3)
The amount of outdoor air and daylight	All items	Far too little (−3)
		Moderately too little (−2)
		Slightly too little (−1)
		Neither too much nor too little (0)
		Slightly too much (1)
		Moderately too much (2)
		Far too much (3)
Reported learning enhancement	All items	Decreased 20% or more (−3)
		Decreased 10% (−2)
		Decreased 5% (−1)
		Neither increased nor decreased (0)
		Increased 5% (1)
		Increased 10% (2)
		Increased 20% or more (3)
GPA	All items	A (1); B (2); C (3)
Daily activity time diary	All items	0 min (0)
		15 min (1)
		30 min (2)
		45 min (3)
		More than 45 min (4)

Fitbit and AWIR data were downloaded directly from the official platforms and are provided in their original, raw formats with no cleaning, transformation, or post-processing.

**Fig. 3:**
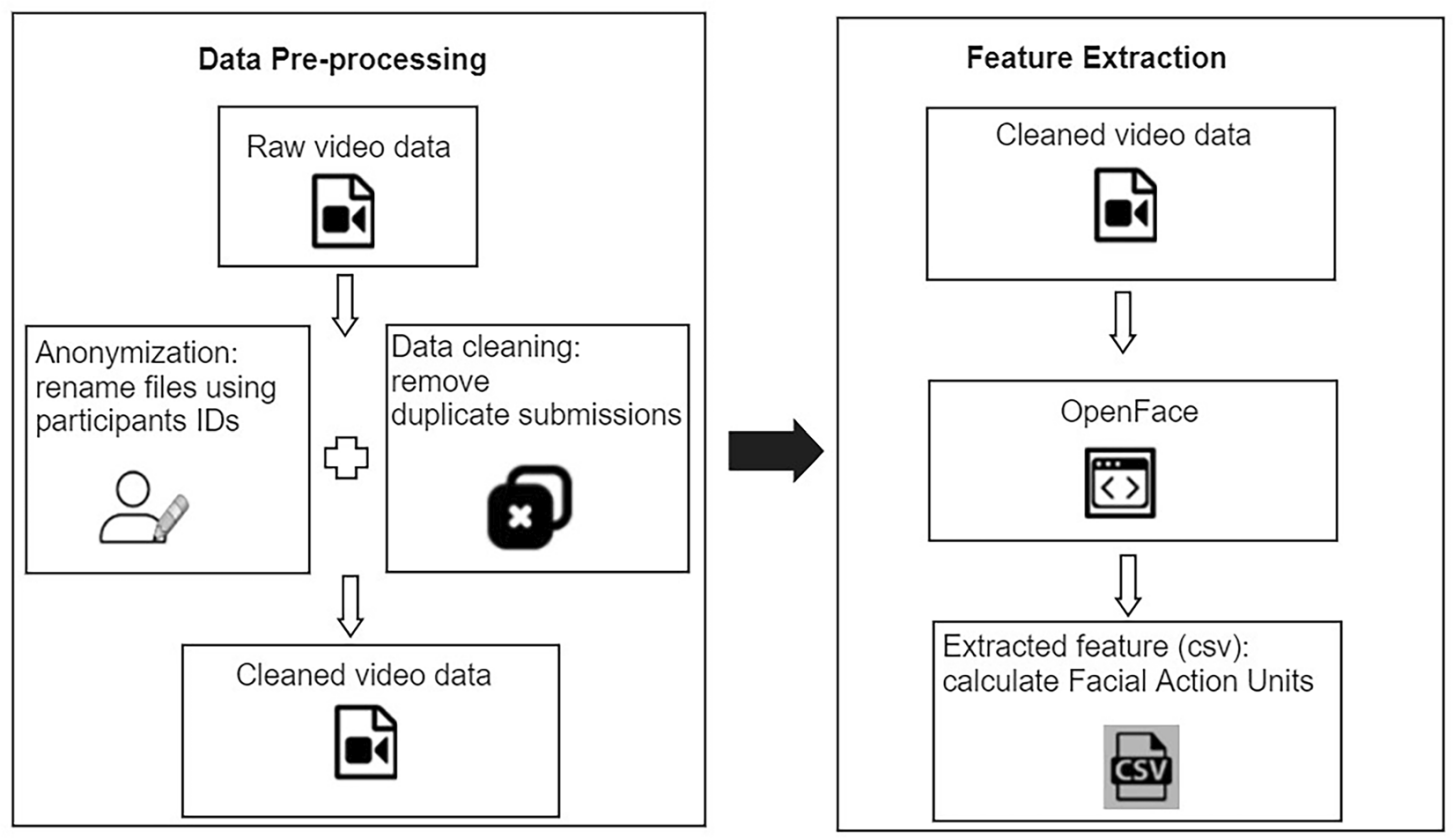
Workflow of the facial expression extraction process. Data was anonymized by renaming files with participant IDs and cleaned by removing duplicate submissions. OpenFace was then used to extract FAUs.

Facial features were extracted from video recordings of students’ (only the participants’) facial expressions during online courses using OpenFace 2.2.0^16^. OpenFace analyzes individual video frames to detect facial landmarks (like eyes, lips, nose, cheeks) and then calculates Facial Action Units (FAUs), which are specific muscle movements corresponding to facial expressions (e.g., an outer brow raise). When OpenFace processes a video and successfully extracts features, it generates a row for each frame with the positions for facial landmarks and action units as columns. However, not all frames were successfully detected, and the failed frames were indicated in the final output data. The detailed video data processing procedure is shown in Fig. 3. For this dataset, only FAUs presence and intensity values were retained. No images, video frames, audio, or facial landmark coordinates were stored or shared.

## Data Records

The files have been deposited in the Harvard Dataverse^17^ and are publicly accessible at: 10.7910/DVN/SJ8ILQ.

### Questionnaire

In this study, all questions were implemented in Qualtrics. The detailed questions in each section have been summarized in the dataset repository.

### Main dataset

**Fig. 4:**
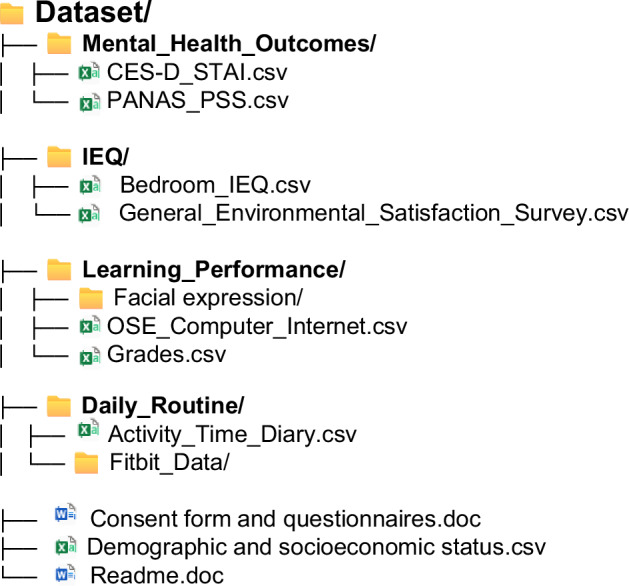
Dataset directory tree. **Mental_Health_Outcomes:** stress, positive and negative affect schedule, depression, and anxiety survey responses; **IEQ:** indoor environmental quality survey data and AWAIR measured data; **Learning_Performance:** extracted facial expression features, OSE survey responses, and GPA data; **Daily_Routine:** daily activity time diary records and raw Fitbit data. Additional supporting documents include the consent form, demographic and socioeconomic data, and the dataset readme file.

The dataset provided consists of four folders, capturing all the data collected during the study. Each folder summarizes data on the same topics. All files include information on ID, date, and the responses to the questions. Detailed content and structure of the dataset is listed in Fig. 4.

## Technical Validation

### Questionnaire data

To ensure the quality and reliability of the questionnaire data, several validation steps were conducted. Internal consistency of the mental health outcome and OSE surveys were assessed using the Cronbach’s alpha test^18^, a standard measure of scale reliability. PSS and the CES-D both demonstrated strong reliability, each with a Cronbach’s alpha of 0.87. The STAI yielded an even higher alpha of 0.95, indicating excellent internal consistency. Similarly, the OSE achieved an alpha of 0.90.

**Fig. 5:**
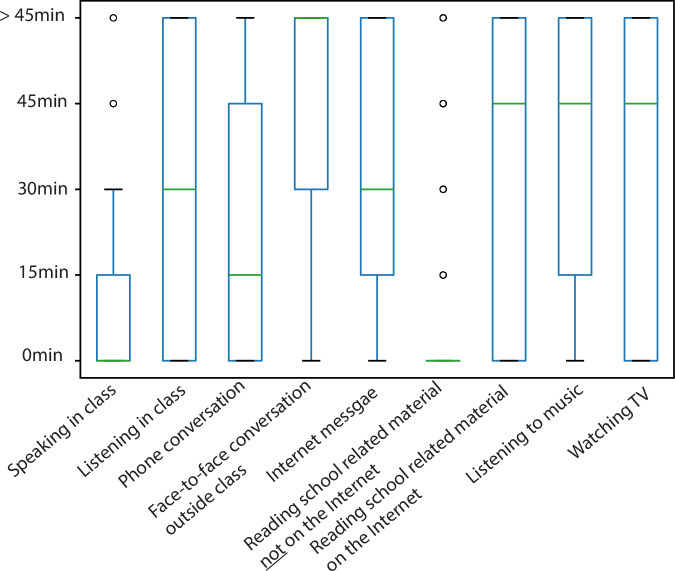
Boxplot of time of daily activity diaries.

Figure 5 shows the distribution of self-reported daily activity diary, which captures participants’ engagement in school-related, social, and entertainment activities. The responses ranged from 0 min to more than 45 min, reflecting time reporting. The boxplots reveal a wide spread of responses across different activity types, indicating variability in participants’ daily routines. Entertainment activities such as “Watching TV” and “Listening to music” show relatively high median values and wide interquartile ranges, whereas cognitively demanding activities like “Reading school-related material not on the Internet” exhibit lower engagement and higher skewness. The detailed time spent diaries of each participant across the cohorts are provided in the dataset.

**Fig. 6:**
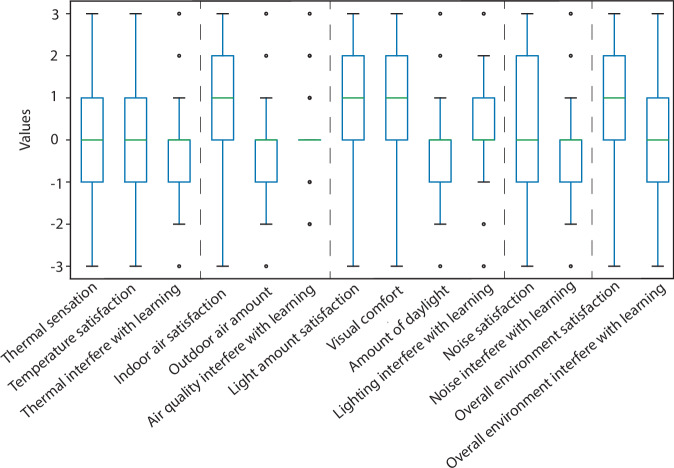
Boxplot of general indoor environment satisfaction survey data. **Thermal sensation** was rated from −3 (cold) to 3 (hot); **Satisfaction variables** were rated from −3 (very dissatisfied) to 3 (very satisfied); **Interference with learning** was coded from −3 (significantly interferes) to 3 (significantly enhances), with 0 indicating no effect; **Outdoor air and daylight amounts** were rated from −3 (far too little) to 3 (far too much); **Reported learning outcomes** ranged from −3 (decreased by 20%) to 3 (increased by 20%).

Figure 6 shows the distribution of participants’ self-reported perceptions of environmental satisfaction and its interference or enhancement with learning. These items were rated on a Likert scale from −3 to +3 as described in Section Data pre-processing. The data show a relatively balanced distribution across categories, with most items exhibiting a reasonable spread.

### Sensor calibration

AWAIR sensors and Fitbits used in this study were brand newly purchased for the study and thus were freshly inspected and calibrated by the manufacturers. Details about the AWAIR sensor’s resolution accuracy are provided in Table 3. The accuracy of Fitbit data is discussed in a review^19^, according to which Fitbit devices are reasonably accurate for step counts but less reliable for energy-expenditure measures. Studies also indicate that Fitbit’s heart rate accuracy at rest is high and comparable to chest straps, Polar monitors, and electrocardiograms (ECG)^20,21^.

**Table 3:** Accuracy of AWAIR sensor data.

Monitored parameter	Device	Resolution	Measurement accuracy
Temperature	AWAIR	0.015 °C	±0.2 °C
CO_2_	AWAIR	1ppm	±75ppm
Relative Humidity	AWAIR	0.01% RH	±2%
TVOCs	AWAIR	1ppb	±15%
PM_2.5_	AWAIR	1 µg/m³	±15 µg/m³

### Quality Control Prior to Feature Extraction

Raw video recordings were used only for feature extraction and are not included in the released dataset to protect participant privacy. A random subset of videos (20%) was manually reviewed to assess image clarity, lighting consistency, and audio quality. This pre-checking step was necessary to ensure that the extracted facial-expression features were reliable and not distorted by poor video quality.

### FAUs Data

To quantitatively assess the video tracking quality, we calculated the frame-level facial feature extraction success rate for each video. Across all videos, the average percentage of successfully detected frames was 87.32%, indicating a generally high tracking quality suitable for downstream analyses.

### Strengths and limitations

This study collected a comprehensive set of multimodal data that integrates Fitbit physiological signals, facial-expressions, indoor environmental monitoring, and self-reported survey responses. Such multimodal data collection aligns with established practices in health and behavioral research, where combining physiological, behavioral, and environmental parameters is recognized for producing richer and more reliable insights than unimodal approaches.

Despite its strengths, this study has several limitations. First, the summer cohort had a smaller sample size than other two cohorts because fewer students enroll in summer courses, which may also limit the robustness of semester-to-semester comparisons. Second, data were collected only during the COVID-19 pandemic; the lack of post-pandemic data restricts our ability to compare changes in students’ mental health status and online learning performance over time. Third, this longitudinal dataset consists of three separate cohorts, and the participants differ across cohorts. This inconsistency may affect the continuity of the time-series data analyses. Finally, because survey questions were not mandatory in order to reduce participant burden and survey dropout, missing data were observed in the demographic and socioeconomic surveys and in academic transcripts. These missing data may not be randomly distributed across demographic or socioeconomic groups or mental health conditions. This limitation should be considered when interpreting the findings and highlights the need for future studies to adopt strategies that better capture demographic information from psychologically vulnerable populations.

## Data Availability

All these files have been deposited in the Harvard Dataverse^17^ and are publicly accessible at: 10.7910/DVN/SJ8ILQ.

## Code availability

No custom code was developed for this dataset. The facial expression features were extracted using OpenFace, an open-source facial behavior analysis toolkit available online^16^.
